# Molecular Dynamic Simulation and Inhibitor Prediction of Cysteine Synthase Structured Model as a Potential Drug Target for Trichomoniasis

**DOI:** 10.1155/2013/390920

**Published:** 2013-09-01

**Authors:** Satendra Singh, Gaurav Sablok, Rohit Farmer, Atul Kumar Singh, Budhayash Gautam, Sunil Kumar

**Affiliations:** ^1^Department of Computational Biology and Bioinformatics, JSBB, SHIATS, Allahabad 211007, India; ^2^Department of Biodiversity and Molecular Ecology, Research and Innovation Center, Fondazione Edmund Mach (FEM), Istituto Agrario San Michele (IASMA), Via Mach 1, 38010 San Michele all'Adige, Italy; ^3^Centre for Research in Nanotechnology and Science, Indian Institute of Technology Bombay, Mumbai 400076, India; ^4^Bioinformatics Centre, Institute of Life Sciences, Nalco Square, Bhubaneswar 751023, India

## Abstract

In our presented research, we made an attempt to predict the 3D model for cysteine synthase (**A2GMG5_TRIVA**) using homology-modeling approaches. To investigate deeper into the predicted structure, we further performed a molecular dynamics simulation for 10 ns and calculated several supporting analysis for structural properties such as RMSF, radius of gyration, and the total energy calculation to support the predicted structured model of cysteine synthase. The present findings led us to conclude that the proposed model is stereochemically stable. The overall PROCHECK G factor for the homology-modeled structure was −0.04. On the basis of the virtual screening for cysteine synthase against the NCI subset II molecule, we present the molecule 1-N, 4-N-bis [3-(1H-benzimidazol-2-yl) phenyl] benzene-1,4-dicarboxamide (**ZINC01690699**) having the minimum energy score (−13.0 Kcal/Mol) and a log *P* value of 6 as a potential inhibitory molecule used to inhibit the growth of *T. vaginalis* infection.

## 1. Introduction

Trichomoniasis has been described as an infection of the urogenital tract; mainly urethra and the vagina are the most common sites of infection in women whereas the urinary tract is the primary site of infection in men. It has been previously reported that the infection usually does not cause symptoms in men [1]. Globally, *Trichomonas vaginalis *is the most prevalent nonviral sexually transmitted infection as per the proposed reports [2, 3]. Since the discovery of the casual agent for the *T. vaginalis *in 1836 [4] and its wide spread infection in women since 1916, several treatments have been made available as a possible medication to control the widespread nonviral transmission. Earlier, metronidazole (1957) was proposed to be the available treatment to which *T. vaginalis *acquired drug resistance in 1962 [5]. Several studies document the fact that it acquires cross-resistance between different nitroimidazoles [6, 7]. Metronidazole is a prodrug, which needs to be activated by enzymes prior to its action on the desired target. In metronidazole-resistant *T. vaginalis*, it was observed that the expression levels of the hydrogenosomal enzymes pyruvate ferredoxin oxidoreductase, ferrodoxin, malic enzyme, and hydrogenase were found to be significantly low, which might probably eliminate the ability of the parasite to activate metronidazole [8, 9]. Development of multidrug resistance strongly justifies the need of the identification of drug targets in *T. vaginalis, *which is a major challenge in modern medicine. With the development of the molecular simulation approaches, novel drug targets of immense potential can be easily identified and can be used as lead molecules for the development of new drug targeted strategies. In order to approach the defense against the protozoan parasite, differences in biochemical mechanisms between parasite and host can suggest promising drug targets without expected cytotoxicity towards the human host. A sequencing effort in 2007 by The Institute of Genomic Research (TIGR) led to the first reports on the draft genome sequence of *T. vaginalis*, which presented us with an open source sequence to look for the wide variations, drug target identification and to hypothesize an effective measure for the effective treatment of the *T. vaginalis *[10].

In the presented research, we aim to concentrate on three hypotheses to postulate the novel drug target for trichomoniasis: (1) to predict the structural model of cysteine synthase, (2) to investigate structural analysis of cysteine synthase using molecular dynamics simulation studies, and (3) to perform virtual screening and docking to identify inhibitor molecules against the suitable ligand. We observed the stable RMSD just after 1 ns time scale. Furthermore, we screened out ten possible ligand molecules after virtual screening of a library of ligands. We found that the ligand **ZINC01690699** has minimum energy score in comparison to the rest of molecules, which have almost the same energy score profile. We demonstrated through our results that **ZINC01690699** showed the most stable hydrogen and hydrophobic interactions with the active site residue as per the Ligplot analysis. We further validated the binding affinity of screened molecules and natural substrate of cysteine synthase using *in silico *docking studies. We conclude that the molecule 1-N, 4-N-bis [3-(1H-benzimidazol-2-yl) phenyl] benzene-1, 4-dicarboxamide (**ZINC01690699**) is proposed to be a potential inhibitor for cysteine synthase. **ZINC01690699** can be further validated for inhibition of *T. vaginalis *by experimental procedures. Our study postulates an important ligand of potential importance for the development of the remedial approaches for the treatment of the *T. vaginalis *infection.

## 2. Materials and Methods

### 2.1. Selection of the Drug Target

*T. vaginalis *is an anaerobic protozoan parasite of humans, which lacks glutathione and relies heavily on cysteine usage as a major redox buffer for the synthesis of the cysteine, a potential molecule of thiol metabolism from sulfide [11–15]. It has been demonstrated earlier that cysteine synthase can be potentially exploited for drug target identification and several drug targets have been identified [11–13]. The metabolic pathway analysis on the possible novel drug target in *T. vaginalis *suggested several drug targets, which can be exploited for the inhibitor designing. Since cysteine synthase is absent in human; therefore, in the present study, we have selected cysteine synthase as the most promising drug target for the evaluation of the molecular dynamics simulations.

### 2.2. Model Generation of the Selected Drug Target

For putative model generation, we retrieved the cysteine synthase (target) amino acid sequence from Uniprot (**A2GMG5_TRIVA**) (http://www.expasy.org) [16]. We found that there is no three-dimensional structure (3D) available for the protein in Protein Data Bank (PDB); hence, we made an attempt to postulate the putative 3D model of the cysteine synthase. To infer the model, we performed BLASTP [17] searches against Brookhaven Protein Data Bank (PDB) [18] with default parameters to find suitable templates for homology modeling. Based on the criteria of maximum identity with high score and lower *e*-value, crystal structure having PDB ID 2BHS was selected as the template for further downstream processing. We observed that the sequence identity and similarity between the target and putative template were 44% and 65%, respectively. Sequence characteristics showed that the target sequence is 299 whereas the template is 303 amino acids long, giving the acceptable query coverage of 97% during the sequence alignment. The sequence alignment of cysteine synthase and the template 2BHS was carried out using the CLUSTALW [19] (http://www.ebi.ac.uk/Tools/msa/clustalw2/) program.

In order to generate the model MODELLER9v6 (http://www.salilab.org/modeller) was used for 3D structure generation based on the obtained information from sequence alignment [20]. The MODELLER software employs probability density functions (PDFs) as the spatial restraints rather than energy [21, 22]. We generated the 3D model of the protein by optimization of the molecular PDFs such that the model violates the input restraints as possible. The molecular PDF was derived as a combination of PDFs restraining individual spatial features of the whole molecule. In our study, MODELLER generated 20 models. The model having the best *G*-score of PROCHECK [23] and with the best VERIFY3D [24] profile was further subjected to energy minimization. VERIFY3D (a structure evaluation server) was used to check the residue profiles of the obtained 3D models. In order to assess the stereochemical qualities of the 3D models, PROCHECK analysis was again performed. Quality evaluation of the model for the environment profile was also predicted using ERRAT (structure evaluation server available at http://nihserver.mbi.ucla.edu/ERRATv2/ [25]). The final refined model was evaluated for its atomic contacts using the WhatIF program [26] to identify the bad packing of side chain atoms or unusual residue contacts. The predicted model was also analysed for error recognition in three-dimensional structure by using ProSA web server [27, 28]. This model was further subjected for the active site identification, molecular dynamics simulations, and docking analysis.

### 2.3. Molecular Dynamics Simulation Study of the Predicted Model

In our study, we performed molecular dynamics (MD) simulations of modeled cysteine synthase protein using GROMACS 4.0.6 software package [29] with GROMOS 96 force field [30] and the flexible SPC water model. The initial structure was immersed in a periodic water box of cubic shape (0.5 nm thick). Electrostatic energy was calculated using the particle mesh Ewald method, which permits the use of Ewald summation at a computational cost comparable to that of a simple truncation method of 10 Å or less [31]. We retained the cutoff distance as 1.0 nm for the calculation of the coulomb and Van der Waal's interaction, respectively. After energy minimization using a steepest descent for 1000 steps, the system was subjected to equilibration at 300 k and normal pressure for 100 ps under the conditions of position restraints for heavy atoms. We subsequently applied LINCS [32] constraints for all bonds, keeping the whole protein molecule fixed and allowing only the water molecule to move to equilibrate with respect to the protein structure. The system was coupled to the external bath by the Berendsen pressure and temperature coupling [33]. The final MD calculations were performed for 10 ns under the same conditions except that the position restraints were removed. The results were analyzed using the standard software provided by the GROMACS package. An average structure was further refined using a steepest descent energy minimization.

### 2.4. Virtual Screening for Validated Model Structure and Ligplot Analysis for Interaction Study

We further performed virtual screening against the NCI subset II molecules retrieved from ZINC database (http://zinc.docking.org) in order to validate the developed molecular structure of cysteine synthase using Autodock Vina (http://vina.scripps.edu/) [34]. In total, 1,364 molecules from the NCI diversity subset II (http://zinc.docking.org/) [35] were screened for the cysteine synthase. Active sites were identified using Putative Active Sites with Spheres (PASS; http://www.ccl.net/cca/software/UNIX/pass/overview.shtml), which uses geometry to characterize regions of buried volume in proteins and to identify positions likely to represent binding sites based upon the size, shape, and burial extent of these volumes. To analyze the docking results, python scripts in MGL tools package were used for visualization. The ten ligands selected based on the energy score after virtual screening were further analyzed by Ligplot program to illustrate the hydrogen and hydrophobic interactions between the ligand and active site residue [36].

## 3. Results and Discussion

*Trichomonas vaginalis, *having anerobic carbohydrate metabolism and devoid of typical mitochondria, is the causative agent of human trichomoniasis [37], a very common sexually transmitted infection with an estimated 170 million cases occurring each year [38], that has been implicated as a major risk factor in predisposition to human immunodeficiency virus/AIDS [39]. The parasite itself is an unusual protozoan that may be one of the earliest branching organisms [40, 41]. It is adapted to an environment containing only low oxygen concentrations by being a fundamentally fermentative organism, with oxygen apparently not making a significant contribution to energy metabolism [42].

### 3.1. Prediction and Validated Modeled Structure of the Cysteine Synthase

In our presented study, we validated the predicted model of cysteine synthase using different validation tools and approaches. Firstly, we evaluated the structure using the Ramachandran plot, and subsequently the structure was analyzed using PROCHECK. We observed that the phi/psi angles of the majority of the residues (92.9%) are present in the most favored regions, followed by 5.5% residues, which were present in the additional allowed regions, whereas 1.2% and 0.4% residues were present in the generously allowed region and in the disallowed regions, respectively. The present findings led us to conclude that the proposed model is stereochemically stable. The overall PROCHECK *G* factor for the homology-modeled structure was −0.04. We observed a decrease in the overall *G* factor after MD simulation, which indicates that there might be an increase in the number of the bad dihedral angles of the modeled structure, which might be possibly due to the MD simulation resulting in an unfavorable dihedral angle, allowing the protein to overcome high-energy barriers. We finally verified the structure using the ERRAT graph estimated using the ERRAT, which analyses the statistics of nonbonded interactions between different atom types. It also gives an estimate of the error value, which is calibrated to estimate the confidence limits of the validated model [25].

We observed a quality factor of 73.592 for the predicted model, which is an indication of the good quality model, as earlier it has been described that models having the quality scores of above 50 are considered as good quality predicted model. The energetic architecture as predicted by PROSA, which gives an estimation of the determination of the native protein fold score, was negative (−9.06) for the modeled protein. The observed value was close to that of template fold score (−9.04), which is a further confirmation of the reliability and stability of the proposed model (Figures 3 and 4). To be accurate in model prediction of cysteine synthase, we further confirmed the model using VERIFY 3D server and WHAT_CHECK. We observed that 80.55% of residues of modeled protein showed a score higher than 0.2, which is a satisfactory 3D-1D score for the residues. On the basis of the above stringent confirmatory tests, we postulate the putative model for the cysteine synthase, which was further used for the virtual screening of the potential inhibitors for cysteine synthase.

**Figure 1:**
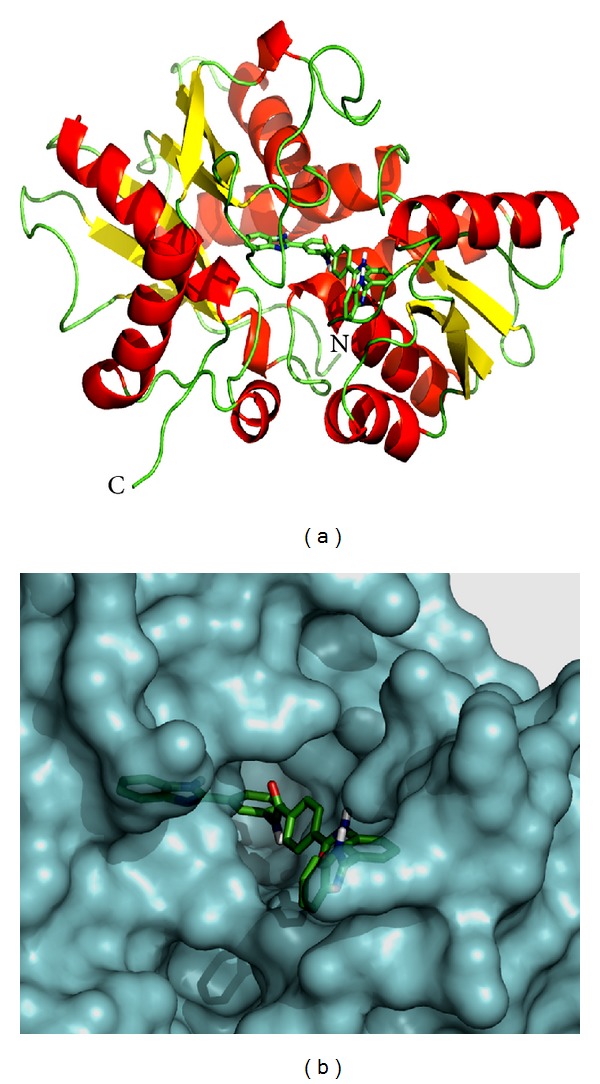
(a) Predicted model of cysteine synthase complexed with ligand **ZINC01690699**. Cysteine synthase is represented in cartoon drawing and ligand in stick form. (b) Modeled cysteine synthase complexed with its best-identified inhibitor **ZINC01690699** in docked form.

**Figure 2:**
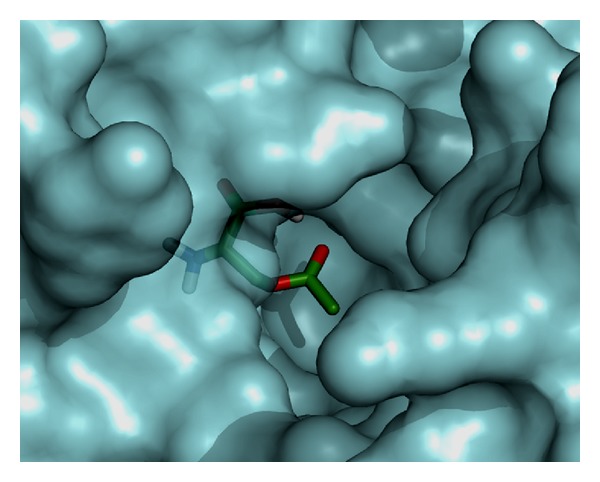
Modeled cysteine synthase complexed with its natural substrate.

**Figure 3:**
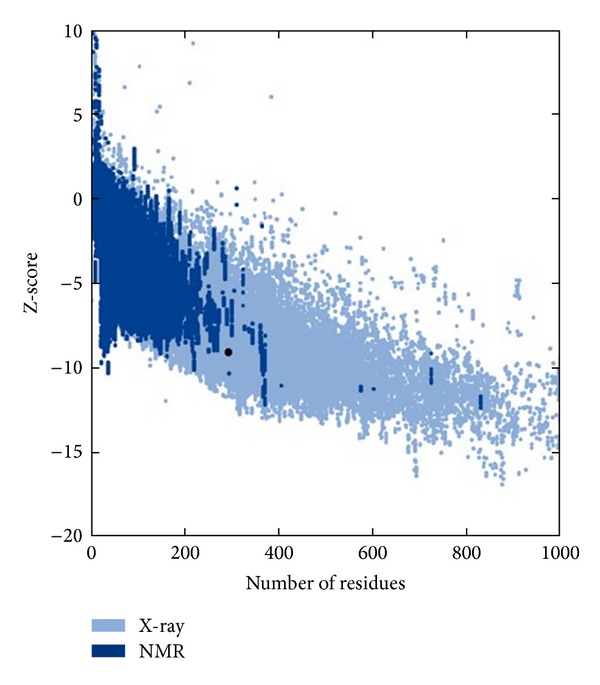
*Z*-score calculated for predicted model of cysteine synthase by PROSA.

**Figure 4:**
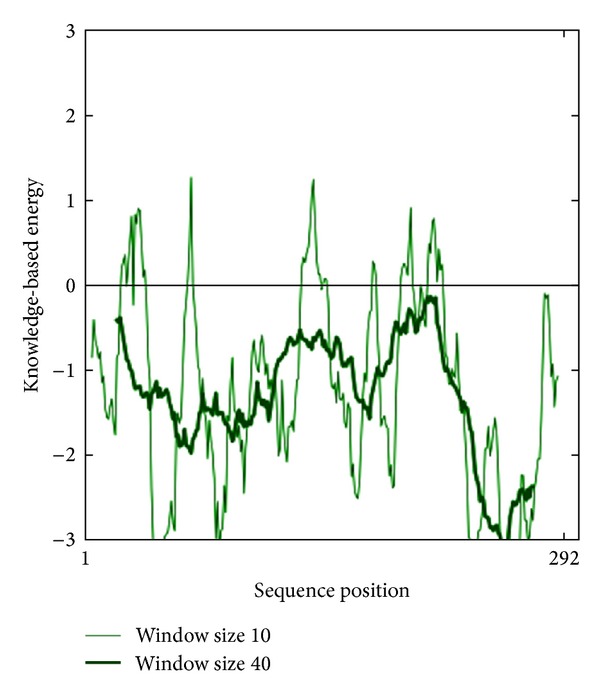
Energy calculated for predicted model of cysteine synthase by PROSA.

The natural substrate, O-acetyl-L-serine, and selected inhibitor molecules after the initial screening were docked with Audodock Vina on cysteine synthase, and structural analysis was conducted as shown in Figure 2. Audodock Vina treats docking as a stochastic global optimization of the scoring function and precalculating grid maps and precalculates the interaction between every atom type pair at every distance. We observed that the docked energy score of cysteine synthase complexed with its best-selected inhibitor molecule **ZINC01690699 (1-N,4-N-bis[3-(1H-benzimidazol-2-yl)phenyl]benzene-1,4-dicarboxamide)** having a molecular weight of 548.606 g/mol was found to be significantly lower (−13.0 Kcal/Mol) as compared to the docked energy score (−5.7 Kcal/Mol) of natural substrate complexed with cysteine synthase (Figures 1(a) and 1(b)). We further evaluated *Z*-score for the overall quality calibration of the model. All the *Z*-scores of the experimentally determined protein chains in current PDB were plotted (Figure 3). In this plot, groups of structures from different sources (X-ray, NMR) are distinguished using different colors where dark blue represents NMR and light blue represents X-ray scores, respectively. The *Z*-score of the predicted cysteine synthase model was found to be within the range of scores, which are typically predicted for native proteins of similar size (https://prosa.services.came.sbg.ac.at/prosa_help.html#output). The energy plot of the predicted model showed local model quality as visualized by plotting energies as a function of amino acid sequence position (Figure 4). We found that the calculated energy for the predicted model of cysteine synthase was negative, which indicates an error free model of the cysteine synthase as positive values correspond to problematic or erroneous parts of the input structure (https://prosa.services.came.sbg.ac.at/prosa_help.html#output).

### 3.2. Molecular Dynamic Simulation of Cysteine Synthase

**Figure 5:**
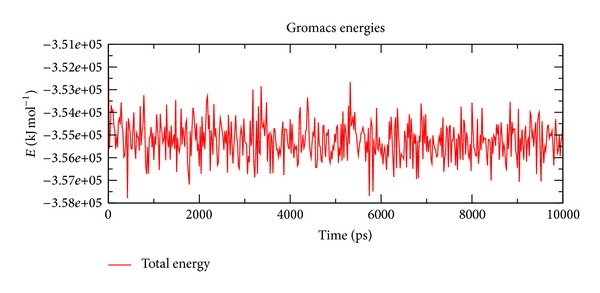
Energy of the molecule during simulation at 10 ns.

**Figure 6:**
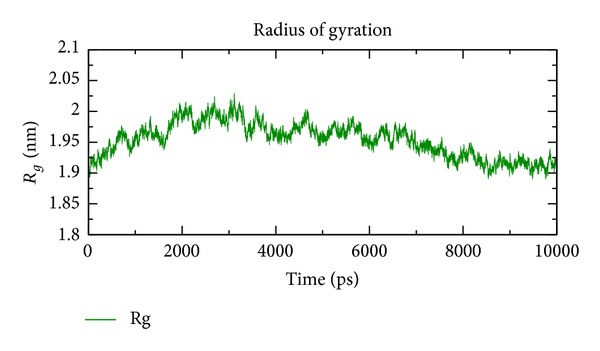
Modeled radius of gyration at 10 ns.

**Figure 7:**
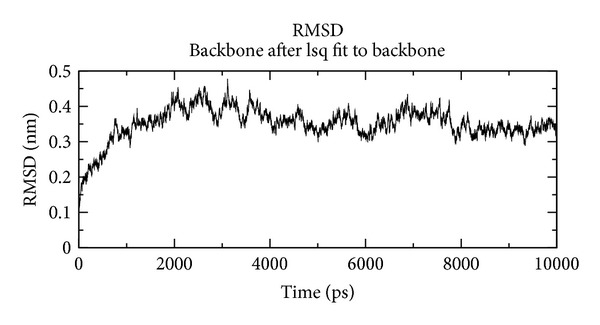
RMSD of modeled structure at 10 ns.

**Figure 8:**
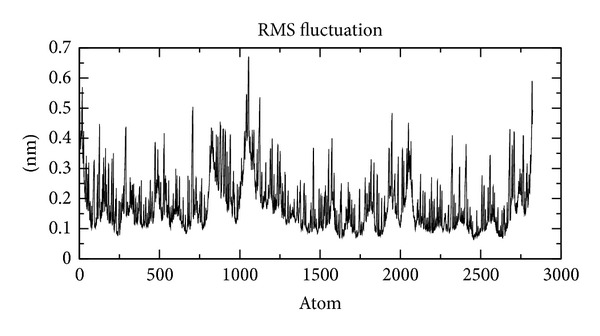
Model RMS fluctuation at 10 ns.

In our study, the predicted model of cysteine synthase has shown good accuracy of the structure as revealed by the virtual screening. It has been earlier described that the virtual screened model should have the stable molecular dynamic behavior. We evaluated the virtual screened model of cysteine synthase using the molecular dynamic stability criteria by performing the molecular dynamic simulation study using GROMACS 4.0.6 software package. In our case, molecular dynamics simulation study revealed a consistent value of the energy of the molecule throughout the time period of simulation, which is an indication of strong basis of the fact that the molecule has a stable structure as required for the virtual screening and drug designing processes (Figure 5). We further evaluated the radius of gyration parameter, and it was observed that initially the radius of gyration was increasing, but after 4000 ps it decreases up to 8000 ps and finally become almost constant for the rest of the duration of the simulation (Figure 6). This observation suggests and is in line with the argument that the cysteine synthase model has a compact structure, which provides the required stability to the molecule and supports our previous molecular dynamic simulations results. Root mean square deviation (RMSD) was evaluated during the simulation, and it was observed that it was increasing in the beginning, but after 2000 ps it became almost constant for the rest of the duration of the simulation, which suggests that the hypothesized cysteine synthase model has lesser RMSD for the cysteine synthase backbone and has less flexibility, indicating the stable dynamic behavior structure of cysteine synthase (Figure 7). It was observed that the fluctuations in the root mean square (RMS) were very low and most of the atoms were free from the RMS fluctuations (Figure 8). Furthermore, there were only few atoms, which showed RMS fluctuation at C and N terminals due to the loop region, which strongly suggests that cysteine synthase model has an accurate and stable structure and can be used for the virtual screening.

### 3.3. Virtual Screening

**Figure 9:**
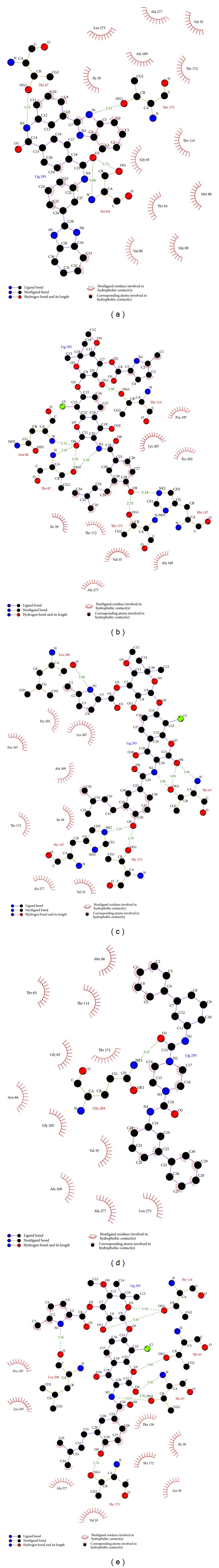
(a) Ligplot for screened ligand **ZINC01690699**. (b) Ligplot for screened ligand **ZINC29590263**. (c) Ligplot for screened ligand **ZINC29590259**. (d) Ligplot for screened ligand **ZINC04783229**. (e) Ligplot for screened ligand **ZINC29590257**.

**Figure 10:**
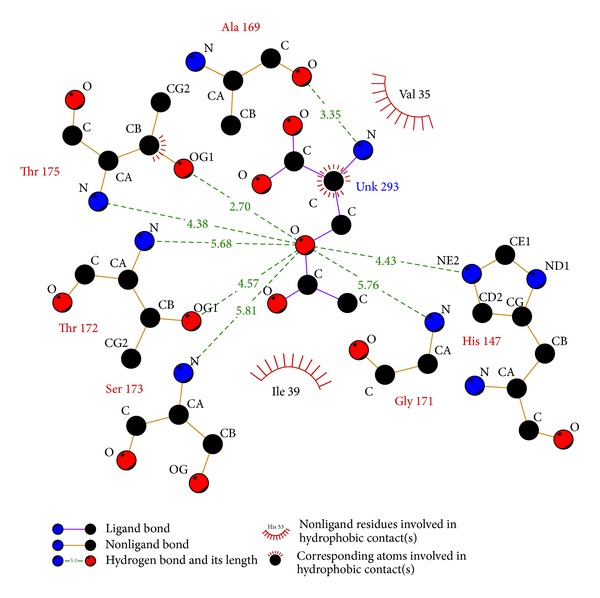
Ligplot of enzyme with its natural substrate O-acetyl-L-serine.

We performed virtual screening of cysteine synthase against the NCI subset II of the ZINC database. The entire top ten molecule hits having the minimum energy were further screened out as the possible inhibitor of the cysteine synthase as shown in Table 1. All the selected molecules have very low energy score (below −11.0 Kcal/Mol) as compared to the energy score of natural substrate with cysteine synthase (−5.7 Kcal/Mol). We observed that three ligands (**ZINC29590263**, **ZINC29590259**, and **ZINC04783229**) possess the same energy score (−11.5 Kcal/Mol) whereas ligands **ZINC13152284**, **ZINC18055497** (−11.0 Kcal/Mol) **ZINC01568793**, and **ZINC01736227** have nearly the same energy score (−11.1 Kcal/Mol). On the basis of the above criteria, we evaluated the interaction kinetics of the observed possible inhibitors. The Ligplot study revealed that among the screened ligands **ZINC01690699** had 12 hydrophobic and 4 hydrogen bond interactions (Figure 9(a)), which is the highest in comparison with the number of interactions as compared to the rest of the molecules (Table 1). On the basis of the high occurrence of the hydrophobic and hydrogen bond interactions, we selected **ZINC01690699** as the possible inhibitor lead molecule as it has minimum energy score and highest number of interactions with the active site residue of the cysteine synthase; as most stable complex molecule as depicted in the interaction profiles between the cysteine synthase and the top five ligands according to the energy score (Figures 9(a) to 9(e) and Figure 10;Table 2). Structure analysis of **ZINC01690699** revealed that it has four hydrogen bonds, which include one bond with each Thr 67 and Thr 175 and two with Ser 64, and twelve hydrophobic bonds, which include Val 35, Ile 39, Thr 63, Gly 65, Met 86, Gly 89, Val 90, Thr 114, Ala 169, Thr 172, Leu 275, and Ala 277, respectively.

Another probable candidate **ZINC29590263** has seven hydrogen bonds for interaction at sites Asn 66, Thr 67, Thr 114, His 147, and Thr 175, respectively. Furthermore, it has eight hydrophobic bonds with amino acids like Val 35, Ile 39, Ala 169, Thr 172, Pro 197, Tyr 202, Lys 207, and Ala 277 (Figure 9(b)). Interaction kinetics of **ZINC29590259** revealed that it has six hydrogen bond interactions with different amino acids at sites Thr 67, His 147, Thr 175, and Leu 206 (Figure 9(c)) whereas total hydrophobic interactions were eight, which include interactions at Val 35, Ile 39, Ala 169, Thr 172, Pro 197, Tyr 202, Lys 207, and Ala 277. We observed that **ZINC04783229** has only one hydrogen bond with Gln 204 whereas it has eleven hydrophobic interactions with different amino acids at sites Val 35, Gly 65, Thr 63, Asn 66, Met 86, Thr 114, Ala 169, Thr 172, Gly 205, Leu 275, and Ala 277, respectively (Figure 9(d)). Another proposed inhibitor **ZINC29590257** has seven hydrogen bonds at Thr 63, Thr 67, Thr 114, Thr 175, and Leu 206 coupled with eight hydrophobic interactions (Val 35, Lys 36, Ile 39, Phe 138, Thr 172, Pro 197, Lys 207, and Ala 277), respectively (Figure 9(e)). Interaction sites of natural substrate O-acetyl-L-serine revealed that it has seven hydrogen bonds with His 147, Ala 169, Gly 171, Thr 172, Ser 173, and Thr 175 and two hydrophobic interactions with Val 35 and Ile 39 (Figure 10). Correlation coefficient analysis was performed between energy scores calculated for all the selected ligand molecules, log *P* value, and molecular weight (Table 1). We observed a negative correlation coefficient between energy score and log *P* value (−0.101) and between energy score and molecular weight (−0.236). Statistical supports as evaluated using the correlations clearly support the fact that the energy score of the ligands is independent of their molecular weight and log *P* values, and energy score may depend on interactions or the conformation of ligands and active site residues. The analysis of the residues taking part in the interactions in the ten selected ligplot molecules and the residues involved in the binding of natural substrate with the cysteine synthase revealed that certain amino acid residues are found to be conserved in most of the analyzed interactions (Table 3). A summary of the hydrogen and the hydrophobic interactions for the top five ligands has been provided (Table 2), which can be potentially exploited as lead molecules for the effective development of docking based targeted drug discovery approaches for *T. vaginalis*.

## 4. Conclusion

*T. vaginalis *is a multidrug resistant pathogen and causes severe infection in humans which has acquired multidrug resistance and cross-resistance against the presently available drugs, such as metronidazole, nitroimidazoles, and tinidazole, which demands the screening of the potential inhibitors for the effective drug administration. In our study, we selected cysteine synthase as the most promising drug target for the drug inhibitor designing. The PDB structure of cysteine synthase was not available; therefore, it was predicted and validated with the help of comparative modeling. The virtual screening for cysteine synthase was carried out against the NCI database using Autodock Vina, which revealed the top five candidates in ZINC database and indicated strong binding affinity for cysteine synthase. We further demonstrated that inhibitor **ZINC01690699,** having the minimum energy score (−13.0 Kcal/Mol) and a log *P* value of 6, possesses the highest binding affinity towards the cysteine synthase and can be a potential putative inhibitor on the basis of the interactions with residues of the active site of cysteine synthase. Our conducted research provides a platform for the experimental verification of the molecule **ZINC01690699** as a potential growth inhibitor of *T. vaginalis*.

## Figures and Tables

**Table 1:** Molecules from NCI subset II of the ZINC database after virtual screening.

S. no.	ZINC ID of the screened molecules	IUPAC Convention of the ligand	Energy score Kcal/Mol	Hydrogen bond	Hydrophobic	Molecular weight (g/mol)	Log *P* value
				Interaction of ligand with active site residues		
1	ZINC01690699	1-N,4-N-bis[3-(1H-benzimidazol-2-yl)phenyl]benzene-1,4-dicarboxamide	−13	4	12	548.59336	6

2	ZINC29590263	8-chloro-7-[(2R,3R,4R,5S)-3-hydroxy-5-methoxy-6, 6-dimethyl-4-(5-methyl-1H-pyrrole-2-carbonyl)oxyoxan-2-yl]oxy-3-[[4-hydroxy-3-(3-methylbut-2-enyl)benzoyl]amino]-4-oxochromen-2-olate	−11.5	7	8	696.12014	6.1

3	ZINC29590259	8-chloro-7-[(2R,3R,4S,5S)-3-hydroxy-5-methoxy-6,6-dimethyl-4-(5-methyl-1H-pyrrole-2-carbonyl)oxyoxan-2-yl]oxy-3-[[4-hydroxy-3-(3-methylbut-2-enyl)benzoyl]amino]-4-oxochromen-2-olate	−11.5	6	8	696.12014	6.1

4	ZINC04783229	1-N,4-N-bis(3-phenylphenyl)piperazine-1,4-dicarboxamide	−11.5	1	11	476.56892	5

5	ZINC29590257	8-chloro-7-[(2R,3R,4S,5R)-3-hydroxy-5-methoxy-6,6-dimethyl-4-(5-methyl-1H-pyrrole-2-carbonyl)oxyoxan-2-yl]oxy-3-[[4-hydroxy-3-(3-methylbut-2-enyl)benzoyl]amino]-4-oxochromen-2-olate	−11.4	7	8	696.12014	6.1

6	ZINC29590262	8-chloro-7-[(2R,3R,4R,5R)-3-hydroxy-5-methoxy-6,6-dimethyl-4-(5-methyl-1H-pyrrole-2-carbonyl)oxyoxan-2-yl]oxy-3-[[4-hydroxy-3-(3-methylbut-2-enyl)benzoyl]amino]-4-oxochromen-2-olate	−11.3	6	8	696.12014	6.1

7	ZINC01568793	2-[4-[4-(1,3-dioxo-2-azaspiro[4.4]nonan-2-yl)-3-methylphenyl]-2-methylphenyl]-2 azaspiro[4.4]nonane-1,3-dione	−11.1	4	6	484.58608	4.7

8	ZINC01736227	(2S)-5-phenyl-2-[(2S)-5-phenyl-2,3-dihydro-1,3-benzoxazol-2-yl]-2,3-dihydro-1,3-benzoxazole	−11.1	2	6	392.4492	6.7

9	ZINC13152284	(2R)-5-phenyl-2-[(2S)-5-phenyl-2,3-dihydro-1,3-benzoxazol-2-yl]-2,3-dihydro-1,3-benzoxazole	−11	3	3	392.4492	6.7

10	ZINC18055497	—	−11	3	3	479.67574	4.7

11	CID_99478 (Natural substrate)	—	−5.7	7	2	147.12926	—

**Table 2:** Calculated hydrogen and hydrophobic interactions for the top five best ranking ligands based on Autodock Vina score and Ligplot interactions.

S. no.	ZINC ID	IUPAC convention of the ligand	Hydrogen bond	Hydrophobic interactions
1	**ZINC01690699**	1-N,4-N-bis[3-(1H-benzimidazol-2-yl)phenyl]benzene-1,4-dicarboxamide	T67, T175, S64	V35, I39, T63, G65, M86, G89, V90, T114, A169, T172, L275 and A277

2	**ZINC29590263**	8-chloro-7-[(2R,3R,4R,5S)-3-hydroxy-5-methoxy-6, 6-dimethyl-4-(5-methyl-1H-pyrrole-2-carbonyl)oxyoxan-2-yl]oxy-3-[[4-hydroxy-3-(3-methylbut-2-enyl)benzoyl]amino]-4-oxochromen-2-olate	N66, T67, T114, H147, T175	V35, I39, A169, T172, P197, T202, K207 and A277

3	**ZINC29590259**	8-chloro-7-[(2R,3R,4S,5S)-3-hydroxy-5-methoxy-6,6-dimethyl-4-(5-methyl-1H-pyrrole-2-carbonyl)oxyoxan-2-yl]oxy-3-[[4-hydroxy-3-(3-methylbut-2-enyl)benzoyl]amino]-4-oxochromen-2-olate	T67, H147, T175, L206	

4	**ZINC04783229**	1-N,4-N-bis(3-phenylphenyl)piperazine-1,4-dicarboxamide	Q204	V35, G65, T63, N66, M86, T114, A169, T172, G205, L275 and A277

5	**ZINC29590257**	8-chloro-7-[(2R,3R,4S,5R)-3-hydroxy-5-methoxy-6,6-dimethyl-4-(5-methyl-1H-pyrrole-2-carbonyl)oxyoxan-2-yl]oxy-3-[[4-hydroxy-3-(3-methylbut-2-enyl)benzoyl]amino]-4-oxochromen-2-olate	T63, T67, T114, T175, L206	V35, K36, I39, P138, T172, P197, K207 and A277

**Table 3:** Composition of active site residues interacting with ligand and natural substrate.

S. no.	Amino acid	Position
1.	Val	35
2.	Ile	39
3.	Thr	63
4.	Asn	66
5.	Thr	67
6.	Thr	114
7.	His	147
8.	Ala	169
9.	Gly	171
10.	Thr	172
11.	Ser	173
12.	Thr	175
13.	Gly	205
14.	Lys	207
15.	Ieu	275
16.	Ala	277
